# HIPEC in Peritoneal Metastasis of Gastric Origin: A Systematic Review of Regimens and Techniques

**DOI:** 10.3390/jcm11051456

**Published:** 2022-03-07

**Authors:** Felix Gronau, Linda Feldbruegge, Frauke Oberwittler, Santiago Gonzalez-Moreno, Laurent Villeneuve, Clarisse Eveno, Olivier Glehen, Shigeki Kusamura, Beate Rau

**Affiliations:** 1 Department of Surgery, Chirurgische Klinik Campus Charité Mitte|Campus Virchow Klinikum, Charité—Universitätsmedizin Berlin, Corporate Member of Freie Universität Berlin, Humboldt-Universität zu Berlin, and Berlin Institute of Health, 13353 Berlin, Germany; felix.gronau@charite.de (F.G.); linda.feldbruegge@charite.de (L.F.); frauke.oberwittler@charite.de (F.O.); 2 MD Anderson Cancer Center, 28033 Madrid, Spain; sgonzalez@mdanderson.es; 3 Réseau National de Prise en Charge des Tumeurs Rares du Péritoine, French National Registry of Rare Peritoneal Surface Malignancies, 69002 Lyon, France; laurent.villeneuve@chu-lyon.fr; 4 Department of Surgical Oncology, CHU Lyon Sud, Hospices Civils de Lyon, 69495 Pierre-Bénite, France; clarisse.eveno@gmail.com (C.E.); olivier.glehen@chu-lyon.fr (O.G.); 5 Peritoneal Surface Malignancies Unit, Fondazione Istituto di Ricovero e Cura a Carattere Scientifico (IRCCS), Istituto Nazionale Tumori dei Tumori di Milano, 20133 Milano, Italy; shigeki.kusamura@istitutotumori.mi.it

**Keywords:** PRISMA, peritoneal metastasis, gastric cancer, intraperitoneal chemotherapy, hyperthermic intraperitoneal chemotherapy (HIPEC), cytoreductive surgery

## Abstract

(1) Background: Peritoneal metastasis in gastric cancer is associated with a poor prognosis. Complete cytoreductive surgery including gastrectomy and complete removal of all peritoneal lesions followed by hyperthermic intraperitoneal chemotherapy (HIPEC) achieves promising results. There exists an immersive variety of approaches for HIPEC that makes it difficult to weigh different results obtained in the literature. In order to enable standardization and development of HIPEC, we here present a systematic review of different drug regimens and technical approaches. (2) Methods: PubMed, Embase, and the Cochrane Library were systematically searched on 26 May 2021 using the mesh terms “intraperitoneal chemotherapy AND gastric cancer”. Under consideration of systematic review guidelines, articles reporting on HIPEC in combination with CRS were selected. Data on duration, drugs, dosage, and other application parameters as well as morbidity and long term survival data were extracted for subsequent statistical analysis, tabulation, and descriptive synthesis. We assessed the risk of bias due to inhomogeneity of the patient cohort and incompleteness of report of HIPEC parameters. (3) Results: Out of 1421 screened publications, 42 publications presenting data from 1325 patients met the criteria. Most of the publications were single institutional retrospective cohort studies. The most common HIPEC regimen is performed after gastrointestinal anastomosis and consists of 50–200 mg/m^2^ cisplatinum and 30–40 mg/m^2^ mytomycin C at 42–43 °C for 60–90 min in a closed abdomen HIPEC system with three tubes. Almost every study reported incompletely on HIPEC parameters. Lower rates of anastomotic leakage were reported in studies that performed HIPEC after gastrointestinal anastomosis. Studies that performed open HIPEC and integrated a two-drug regimen indicated better overall survival rates. (4) Discussion: This is an exhaustive overview of the use of drug regimens and techniques for HIPEC after CRS for gastric cancer peritoneal metastasis. Other indications and application modes of intraperitoneal chemotherapy such as prophylactic or palliative HIPEC apart from CRS were not addressed. (5) Conclusion: Complete report of HIPEC parameters should be included in every publication. A consensus for dose expression either per BSA or as flat dose is desirable for comparison of the drug regimens. Despite numerous variations, we identified the most common regimens and techniques and their advantages and disadvantages according to the data in the literature. More phase I/II studies are needed to identify the best approach for HIPEC. (6) Other: This review was not supported by third parties.

## 1. Introduction

Despite recent efforts in prevention and early detection, gastric cancer (GC) still remains one of the most common and lethal neoplastic diseases worldwide, accounting for more than one million new cases in 2020 and 7.7% of all cancer-related deaths [1]. 

The peritoneum represents the second most common site of gastric cancer metastasis and is the most common site of cancer recurrence [2,3]. Peritoneal metastasis of gastric cancer (pmGC) has very poor median survival rates of only 3–6 months [4,5,6]. Palliative systemic intravenous chemotherapy still represents the standard treatment strategy for pmGC.

Hyperthermic intraperitoneal chemotherapy (HIPEC) combines the concept of direct delivery of the chemotherapeutic agent to the peritoneum, enabling the application of higher local doses with low systemic toxicity and the enhancement of its cytotoxic effects using hyperthermia [7,8,9]. HIPEC even offers the possibility of cure for a highly selected cohort of patients in cases of complete surgical resection of all peritoneal metastases and simultaneous oncologic gastrectomy with tumor-free resection margins and D2-lymphadenectomy [10,11].

HIPEC has proven promising survival outcomes in many tumor entities such as peritoneal mesothelioma [12], pseudomyxoma peritonei [13], and ovarian cancer [14], with acceptable morbidity and mortality rates and costs [15,16]. Promising results were achieved for pmGC with different technical and drug regimens of HIPEC [17]. 

Although the standardized use of HIPEC in the treatment algorithm of pmGC has not yet been integrated in national and international guidelines, CRS and HIPEC are performed worldwide with an immersive variety of technical and pharmacological approaches. On this account, it is still difficult to compare the results of both the existing randomized controlled studies (RCTs) and retrospective cohorts [18]. Kusamura et al. have identified eight aspects that can be influenced when performing HIPEC: type, combination and concentration of drugs, carrier solution and its volume, temperature, duration, and technique (open or closed abdomen) [19], leaving countless possibilities for pharmacological studies on HIPEC. Recently, standardization of HIPEC has been identified as an important element in order to find the ideal regime and technical approach and to further test the survival benefit intraperitoneal chemotherapy might offer for pmGC [20,21,22].

In an effort to contribute to this standardization of HIPEC regimens, we present a systematic review of all existing RCTs and prospective and retrospective trials with respect to technical approaches and drugs used for HIPEC in the context of CRS for pmGC. 

## 2. Materials and Methods

### 2.1. Literature Search and Selection of Records

This review was conducted taking into account the 2020 PRISMA guidelines for systematic reviews [23]. with respect to different nominations of HIPEC in the past [24] and without limiting the publication date, we systematically searched PubMed, Embase, and the Cochrane Library on 26 May 2021 using the search terms “intraperitoneal chemotherapy AND gastric cancer” as MeSH Terms, as these terms represent the widest definition of intraabdominal locoregional chemotherapeutic drug therapy for gastric cancer peritoneal metastases and include the following translations:

- intraperitoneal: “intraperitonal” [All Fields] OR “intraperitonally” [All Fields] OR “intraperitoneal” [All Fields] OR “intraperitoneally” [All Fields]
- chemotherapy: “chemotherapy’s” [All Fields] OR “drug therapy” [MeSH Terms] OR (“drug” [All Fields] AND “therapy” [All Fields]) OR “drug therapy” [All Fields] OR “chemotherapies” [All Fields] OR “drug therapy” [Subheading] OR “chemotherapy” [All Fields]
- gastric cancer: “stomach neoplasms” [MeSH Terms] OR (“stomach” [All Fields] AND “neoplasms” [All Fields]) OR “stomach neoplasms” [All Fields] OR (“gastric” [All Fields] AND “cancer” [All Fields]) OR “gastric cancer” [All Fields]

After exclusion of other species than human and other languages than English, French, or German, 828 publications were eligible for title, abstract, and full-text screening, which was performed independently by F.G. and F.O. without the use of an automation tool. In case of a discordance between F.G. and F.O., B.R. and L.F. helped to solve the conflict by discussion.

We limited our analysis to studies that performed HIPEC directly after cytoreductive surgery. For this reason, we excluded studies performing HIPEC without CRS or for cytology-positive GC, as well as early postoperative intraperitoneal chemotherapy (EPIC) and neoadjuvant intraperitoneal and/or systemic chemotherapy (NIPS). Further exclusion criteria were introduction of intraperitoneal chemotherapy via direct single or multiple injections or an implanted port-system without an in- and outflow perfusion system, as these procedures are normally performed separately from CRS as well [25]. Articles presenting the results of patient cohorts including multiple tumor entities were excluded when the GC cohort comprised fewer than 6 patients, as we considered such small groups as not representative. We excluded publications when there was no information available on the drug regimen used for HIPEC for pmGC. We considered this aspect crucial for this systematic review. We did not include data from other reviews.

In cases of multiple publications from the same group, we selected the newest or the one with the most complete patient cohort. 

**Figure 1:**
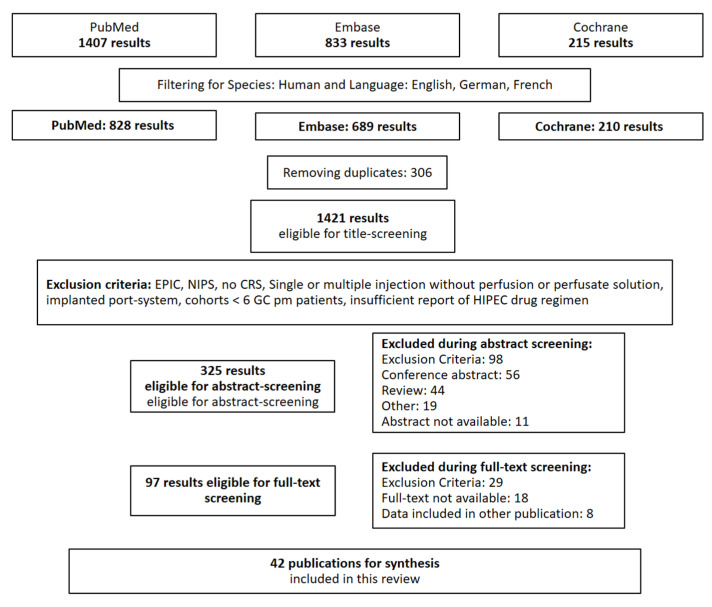
Methodology for article screening.

To identify study protocols for RCTs testing HIPEC for pmGC, we included the study protocols identified during the article screening and performed a PubMed search using the terms “study protocol AND hipec AND gastric cancer” as well as a search in clinicaltrials.gov using the terms “gastric cancer peritoneal metastasis”, “gastric cancer stage iv”, “intraperitoneal chemotherapy”, “HIPEC”, and “peritoneal metastasis”. We adopted the same exclusion criteria as for the already published studies and further only included phase III studies that have not published their results yet (see Figure 1).

### 2.2. Quality Assessment

As we did not intend meta-analysis or other forms of comparative statistical analysis of the outcome data obtained with each drug regimen or technical approach, we did not perform a quality assessment of each study with a PICO (patient cohort, intervention, comparator, outcome)-based tool such as RoB-2 or ROBINS-I [26,27]. Instead, our goal was to provide an exhaustive overview of regimens and techniques used for HIPEC for pmGC and the respective morbidity and survival results. We therefore assessed each publication for the homogeneity of the patient cohort with regard to tumor entity (gastric cancer) and stage (peritoneal metastasis), as well as completeness of report of HIPEC parameters, as we deemed these parameters crucial for an exact overview of adapted techniques. A completed table with a quality and risk of bias assessment due to missing data is attached in the supplements (Table S1).

### 2.3. Data Extraction

F.G. and F.O. independently extracted the data. In cases of a discrepancy during data extraction, again L.F. and B.R. helped to solve the conflict by discussion. We systematically extracted the median PCI, sequence of HIPEC before or after gastrointestinal anastomosis, duration in minutes, open or closed technique, maximum heat, drug regimen including drug name, sequence of application and dose, perfusate solution and volume, flow rate, number of in- and outflow tubes, and the use of simultaneous bidirectional intravenous chemotherapy. The following outcome data were extracted, as well the rate of anastomotic leakage, median overall survival (median OS), median PCI, and major complication rate ≥ III° according to the Clavien–Dindo classification [28]. If only the abstract was available or information was missing, we did not contact the authors.

### 2.4. Data Synthesis

The data were synthesized in a table, and basic statistics were calculated using Microsoft Office (Microsoft, Redmond, WA, USA) and SPSS (Version 25.0. IBM Corp., Armonk, NY, USA). All information collected from the studies can be found in Table 1, Table 2 and Table 3 in this review. For each parameter, the range and distribution of frequency is displayed in the text, as well as the number of missing values. If there were different technical or drug regimens used in one study, all were included in the synthesis, and if available, the percentage of patients treated in each approach was indicated. Concerning the outcome data, the median and the range were compared between the different subgroups and regimens in order to describe the given differences in the literature.

## 3. Results

### 3.1. Publications on CRS and HIPEC for pmGC

The oldest publication found in the initial search dates from 1957, the latest from 25 May 2021. In total, 42 publications published between 1988 and 2021 were included in this review, including 2 RCTs, 5 phase I/II clinical trials, two prospective cohort studies, and 33 retrospective studies reporting on 1325 patients treated with CRS and HIPEC for pmGC. Most of the studies were conducted as single-center studies, five publications presented the results of multi-center cohorts (see Table 1).

### 3.2. Study Protocols

Three ongoing phase III studies met our inclusion criteria. All of them were multi-centric and measured the long-term survival as a primary endpoint. The Chinese trial conducted by Li et al. was a one-armed study that performed closed HIPEC with 12 mg docetaxel in 5 L saline for 70 min at 43 °C. Rau et al. compared CRS only with CRS and HIPEC in the GASTRIPEC trial. They used mitomycin C 15–30 mg/m^2^ and cisplatin 75–150 mg/m^2^ in a maximum of 5 L perfusion. The PERISCOPE II trial by van Sandick et al. compared CRS and HIPEC with oxaliplatin 460 mg/m^2^ at 42 °C for 30 min followed by docetaxel 50 mg/m^2^ at 37 °C for 90 min in the intervention arm with standard palliative chemotherapy in the control arm. For more details of the study protocols please refer to Table 2.

### 3.3. Open or Closed HIPEC before or after Anastomosis 

For an overview on the technical parameters of HIPEC that were utilized in each publication, please refer to Table 3. Most of the groups performed HIPEC after gastrointestinal anastomosis (17). However, this important information was not included in 38% of publications (16 studies). Nine groups reported to have started HIPEC before completion of anastomosis. Three of them reported anastomotic leakage rates ranging from 1% to 22% (median: 13%), which is higher than the 0% to 6.5% (median: 0%) rate indicated by 9 groups that performed HIPEC after gastrointestinal anastomosis. HIPEC after closure of the abdomen was more common than open HIPEC (17 vs. 13 publications), although five trials reported the use of both techniques and seven publications did not report on this parameter. Seven studies reported outcome results for their pmGC patients treated with CRS and closed HIPEC, ranging between 6.1 and 33.8 months (median: 11.75 months), which was lower than the median survival of 15 months stated by seven groups that performed open HIPEC (range: 8.9–21.2 months). However, this tendency should be carefully interpreted, as the patient cohorts might be completely different, and the median PCI was not available in most of the underlying studies.

### 3.4. Duration of HIPEC, Temperature, and Choice of Chemotherapeutic Regimen

**Figure 2:**
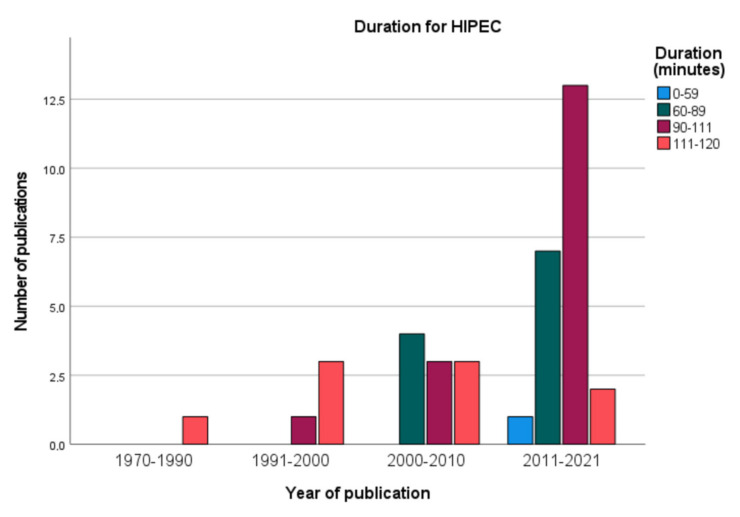
Duration for HIPEC.

HIPEC perfusion lasted for 30 to 120 min within the peritoneal cavity. There has been a trend over the years towards longer durations of HIPEC, as Figure 2 shows. The overall median duration in the underlying publications was 90 min, which was reported by eleven studies. Ten studies used a duration of 60 min and nine studies reported various durations depending on the regimen that was used. Exposure time was shortened to 30 min for application of oxaliplatin in five studies, while MMC was applied for up to 120 min in seven studies. Only four publications did not mention the duration for HIPEC. 

The median temperature of HIPEC in the underlying studies was 42.5 °C; 26 studies reported temperatures between 42 and 43°. Five earlier publications describe a maximum heat of 45–49 °C, while in eight publications, temperatures below 42° were used. Four studies did not report on the applied temperature. If docetaxel was part of the HIPEC regimen as in one study, the inflow temperature was decreased to 37 °C, as taxanes do not necessitate heat activation [61,63].

**Figure 3:**
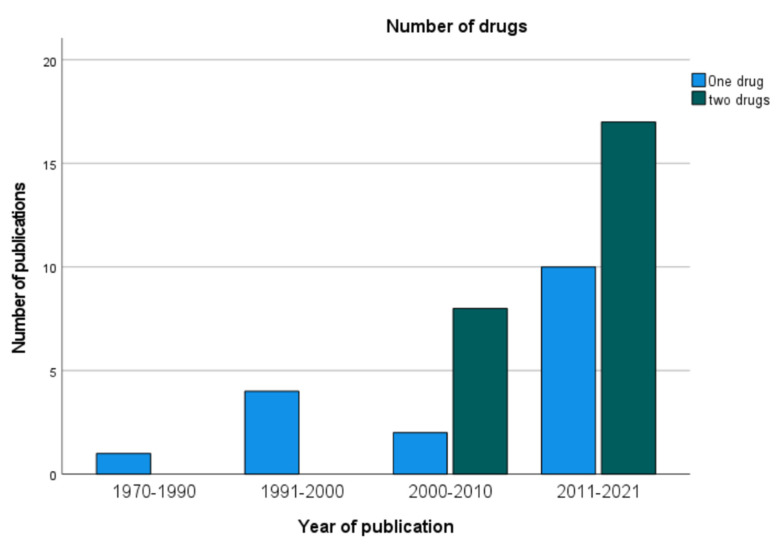
Number of drugs used for HIPEC.

In 54.1% of the studies, two chemotherapeutic drugs were used for HIPEC, mostly a combination of MMC and cisplatin (CDDP), as in 11 studies. Other duplet combinations were CDDP and doxorubicin (6 studies) and oxaliplatin combined with either irinotecan or docetaxel (both in 2 studies). Monotherapy was less common (19 studies) and consisted in 14 studies of MMC, which was part of 30 regimens in total. Figure 3 depicts the development and growing importance of a duplet drug regime since the 2000s. 

**Figure 4:**
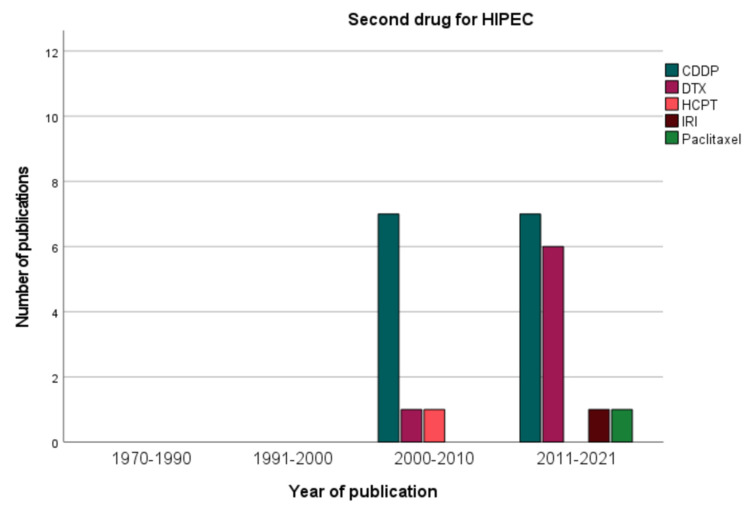
Second drug for HIPEC.

**Figure 5:**
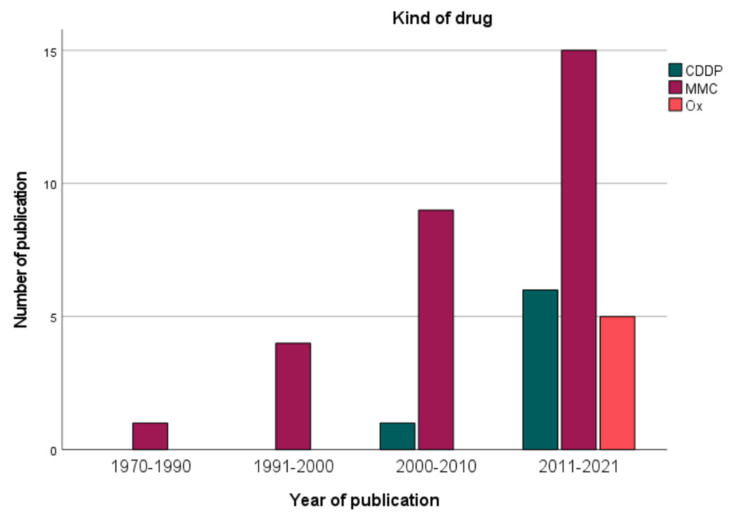
Kind of drug.

Among the groups that used two drugs for HIPEC, the median survival was reported by 10 groups and ranged between 9.2 and 21.2 months (median: 15 months). The median survival after mono-drug HIPEC seems to be lower and was indicated in 11 studies with a range of 6.1 to 33.8 months (median: 11 months), but median PCI was rarely stated for the pmGC patients. Oxaliplatin (OX) or CDDP monotherapy was used in 4 studies and in 3 studies, respectively. CDDP was part of the HIPEC regimen in 20 studies in doses between 50 and 200 mg/m^2^ body surface (median: 75 mg/m^2^), mostly in combination with other drugs, as Figure 4 shows. Dosage for MMC was ranging between 10 and 120 mg/m^2^, for OX between 200 and 460 mg/m^2^ (median: 460 mg/m^2^), and for doxorubicin between 15 and 50 mg/m^2^. Some studies assessed the maximum tolerated dose of irinotecan or doxorubicin, which is more of a trend, as Figure 4 illustrates. Four groups simultaneously applied intravenous 5-fuorouracil with or without leucovorin to enhance cytotoxicity of intraperitoneal oxaliplatin [22]. Figure 5 depicts the evolution of chemotherapy regimens for HIPEC. While the first studies used MMC, CDDP was introduced in the 2000s and oxaliplatin in the last decade. The dosage was difficult to compare between the studies, as some studies did not express the dose per BSA but as a flat dose in total or even per liter of perfusion solution.

### 3.5. Number of Tubes, Perfusate, and Flow Rate

Authors rarely described the placement as well as the numbers of tubes in the abdominal cavity for in- and outflow of the HIPEC perfusion. Only in 43.9% reported this aspect. Most commonly, as in six publications, respectively, two or three tubes in total were used. Three studies described the use of four or five tubes in total. Most groups (*n* = 23) did not report the type and volume perfusion solution that they used; however, saline (8 studies) was the most common one. Glucose-containing solution was used in three studies, as oxaliplatin necessitates chloride-free solutions [69]. The volume was ranging between 2 and 12 L, with a median of 4 L (eight studies). Only 38.1% (16) of the underlying publications reported the flow rate of HIPEC perfusion, with a median around 500 mL/min, as stated in six studies. A higher flow rate up to 10 L/min was applied in seven studies, a lower flow rate between 200 and 400 mL/min in four studies. 

## 4. Discussion

Our aim was to collect the latest literature from both prospective and retrospective trials and to systematically analyze the given data on the technical approaches, therapy protocols, drug, and parameter selection. 

With an immersive variation, HIPEC attracts a lot of attention worldwide. In the 42 publications that were analyzed in this systematic review, every group used a different approach and drug regimen. However, closed HIPEC was more common than open, with most groups performing HIPEC after suture of gastrointestinal anastomosis. 

The delivered temperature ranged from 40 to 49 °C but mostly around 43 °C and measured mainly intra-abdominally. Temperatures differed due to the number and localization of measurement points. This made interpretation of results difficult.

HIPEC drug regimens preferred CDDP and MMC as a duplet regimen, which was most frequently used, although the dosages varied greatly. In this context, the different outcome results are difficult to weigh.

Open HIPEC and duplet therapy according to the data reported in this literature review might lead to an improved survival. Open HIPEC as described by Sugarbaker et al. offers the theoretical advantage of a uniform distribution of heat and liquid chemotherapy [70]. It also allows direct agitation of the chemotherapeutic solution by the surgeon and continuous visualization of the small bowel in order to identify possible penetrations [71]. However, more heat is necessary to maintain hyperthermia, and there is a potential higher risk for the operating staff than with the closed HIPEC [71]. The closed HIPEC in contrast allows an increased intra-abdominal pressure that may lead to an increased tissue penetration of the chemotherapy [71]. Addressing the number of in- and outflow catheters in vivo studies show that four inflow tubes might lead to a more stable and uniform hyperthermia within the abdominal cavity [72]. Nevertheless, several unknown confiders such as PCI, underlying localization of gastric cancer, completeness of cytoreduction, etc. make interpretation difficult and should be discussed with caution (Table 4).

Anastomotic leakage rate was reported higher in publications where gastrointestinal anastomosis was performed after HIPEC. This aspect was discussed in only a few publications. Leiting et al. retrospectively analyzed outcome parameters in a cohort of 1812 patients with mostly colorectal and appendiceal peritoneal metastases undergoing CRS and HIPEC. He found neither the open nor the closed technique to be an independent risk factor for post-operative complications or inferior long-term outcome [73], although others have detected decreased cardiac index, hepatic blood flow, and liver function due to the high abdominal pressure in closed abdomen HIPEC [74]. Somashekar et al. recently compared patients undergoing bowel anastomosis before and after HIPEC and found no significant difference in leakage or perforation rate between the two groups, but in this cohort only 7% pmGC patients were included [75]. 

Concerning the choice of drug for HIPEC, Woeste et al. have compared MMC and OHP for colorectal cancer peritoneal metastases. The choice of drug was not predictive of overall survival [76]. At the same time, three RCTs comparing intravenous OHP and CDDP for gastric cancer were compared in a meta-analysis by Montagnani et al., with a survival benefit and less adverse events in favor of OHP [77], possibly indicating a benefit in intraperitoneal delivery. The ideal drug for intraperitoneal chemotherapy shows a high concentration in the peritoneum, with a high penetration depth into the cancer nodule on one hand, and on the other hand, slow diffusion beyond the peritoneum blood membrane resulting in reduced systemic uptake in order to prevent systemic toxicity [22]. For DTX and OX, it was recently shown in pmGC patients undergoing CRS and HIPEC that systemic uptake is low after intraperitoneal application [78], while this has longer been known for CDDP, doxorubicin, and MMC [71,79]. 

CRS and HIPEC with intraperitoneal oxaliplatin at a dosage of 460 mg/m^2^ for 30 min at a temperature of 43 °C, as performed in the PRODIGE 7 trial, did not show significant benefit compared with CRS alone in peritoneal metastatic colorectal cancer [80]. However, pretreatment with oxaliplatin-containing systemic regimens reduces the sensitivity of colorectal cancer cells [81]. Additionally, low systemic uptake oxaliplatin is associated with postoperative hemorrhage [82], and cisplatin is associated with nephrotoxicity [83], which are both dose-limiting adverse effects. Concerning the nephrotoxicity, it will be interesting to see, whether sodium-thiosulfate, which has recently been shown to significantly reduce acute kidney injury [84], might allow increased intraperitoneal doses of cisplatin. 

A handful of systematic reviews and meta-analyses addresses the indications and results of HIPEC in the context of cytoreductive surgery for gastric cancer peritoneal metastasis [85,86,87,88]. Granieri et al. performed a systematic review and meta-analysis of 12 randomized controlled trials in 2021 [89]. Eveno and Pocard reviewed the literature on this issue in 2016 and, as have other reviews, they only included data from randomized controlled trials [87,90,91] or did not focus on the different protocols for HIPEC [92,93]. 

The variations in different drug regimens and protocols have also been studied for colorectal cancer by Yurttas et al. [94]. Braam et al. reviewed the literature in June 2013 for different chemotherapeutical regimens in intraperitoneal chemotherapy delivery for pmGC [95]. Heldermann systematically reviewed different techniques used for intraperitoneal chemotherapy in general, but included only 29 publications until 2019 [96]. 

Brandl et al. recently presented a review concentrating on different therapeutic regimens of intraperitoneal chemotherapy, including NIPS, EPIC, and abdominal access port-based intraperitoneal chemotherapy. However, they did not include any study protocols of unpublished studies or retrospective cohorts with fewer than 50 patients, which represent 82.5% of the published studies we included in our analysis [25]. 

We think that the depicted differences in the regimens and outcomes should encourage further clinical trials on different HIPEC parameters, such as open vs. closed HIPEC before or after gastrointestinal anastomosis and more dose-finding phase I and II studies in order to identify the best approach for HIPEC in the treatment of pmGC. 

## 5. Conclusions

This is the most current and comprehensive systematic overview of drug regimens and technical approaches for HIPEC that were used in the literature from 1988 to 2021. We here presented not only the most common HIPEC parameters but also the results achieved with the respective approaches. However, due to the great number of retrospective analyses, small number of patients, and lack of reported patient characteristics (for instance: no documentation of PCI in 25 of the 42 discussed papers (59.5%)) we only focused on HIPEC delivery. 

The most common HIPEC regimen for pmGC consists of CDDP 75 mg/m^2^ and MMC 30–40 mg/m^2^ dissolved in 4 L of saline solution applied after suture of gastrointestinal anastomosis at 42–43 °C for 60–90 min via three tubes with a flow rate of 500 mL/min in a closed abdomen HIPEC system. Open HIPEC and two-drug regimen showed better survival results according to the literature, but its use should be weighed with caution. HIPEC after gastrointestinal anastomosis reduces the leakage rate. Standardization of technical aspects of HIPEC such as open or closed abdomen, perfusate, number of tubes, temperature, and duration might help when comparing different drug regimens and might have an influence on survival and morbidity. Further comparison of technical approaches and different drug regimens in phase I/II trials are needed in order to identify the best approach for pmGC. 

## Figures and Tables

**Table 1:** Full list of published studies included.

Reference	Year	Single- or Multi-Institutional	Type of Study	Number of Patients Included	Number of Patients with pmGC and CRS + HIPEC
Fujimoto [29]	1988	single	PCS	15	9
Fujimoto [30]	1997	single	RCS	48	30
Chen [31]	1997	single	RCS	42	6
Sayag-Beaujard [32]	1999	single	pCTII	83	18
Loggie [33]	2000	single	pCTII	84	22
Glehen [34]	2004	single	RCS	49	21
Yonemura [35]	2005	single	RCS	107	42
Kusamura [36]	2006	single	RCS	209	12
Roviello [37]	2006	single	RCS	59	6
Scaringi [38]	2008	single	RCS	37	26
Yang [39]	2009	single	RCS	21	12
Piso [40]	2009	single	RCS	37	11
Yang [41]	2010	single	pCTII	28	28
Li [42]	2010	single	RCS	128	10
Glehen [43]	2010	multiple	RCS	159	147
Yang [44]	2011	single	RCT	68	34
Cotte [45]	2011	single	pCTI	12	12
Mizumoto [46]	2012	single	RCS	250	16
Wu [47]	2013	single	RCS	62	32
Yarema [48]	2014	single	RCS	98	20
Tabrizian [49]	2014	single	RCS	170	12
Rudloff [50]	2014	single	RCT	17	9
Magge [51]	2014	single	RCS	23	23
Levine [52]	2014	single	RCS	1000	46
Kim [53]	2014	single	RCS	112	9
Königsrainer [54]	2014	single	RCS	18	13
Graziosi [55]	2014	single	RCS	36	15
Polanco [56]	2015	single	PCS	370	24
Desantis [57]	2015	single	RCS	356	14
Wu [58]	2016	single	RCS	50	50
Kopanakis [59]	2017	single	RCS	14	14
Rihuete Caro [60]	2018	single	RCS	35	32
Montori [61]	2018	single	RCS	150	26
Yarema [62]	2019	multiple	RCS	117	70
Solomon [63]	2019	single	RCS	268	18
Rau [4]	2019	single	RCS	88	58
Manzanedo [64]	2019	multiple	RCS	88	84
Kimbrough [65]	2019	multiple	RCS	28	28
Hotopp [66]	2019	single	RCS	26	26
Bonnot [17]	2019	multiple	RCS-PSm	275	180
Braeuer [67]	2020	single	RCS	109	37
Koemans [68]	2021	single	pCT I–II	25	23

PCS: prospective cohort study; RCS: retrospective cohort study; RCT: randomized controlled trial; pCTI: prospective clinical trial phase I; pCTII: prospective clinical trial phase II; PSm: propensity score matched.

**Table 2:** Study protocols for phase III studies evaluating CRS and HIPEC with unpublished results.

NCT Number	Patients	End Until	Location	Arm Intervention	Arm Control	Open/Closed	HIPEC Drug	HIPEC Solution/Duration	Temperature	Primary Outcome Measures	Secondary Outcome Measures
NCT03023436	220	22 June	China	CRS + HIPEC + sCTx	single arm	closed	DTX 120 mg	5 L saline; 70 min	43 ± 0.5 °C	MS 2-year (24 months)	1. 2-year OS; 2. 2-year PFS; 3. M&M (30 d; 24 months)
NCT02158988	105	21 September	Germany	CRS + HIPEC + sCTx	CRS + sCTx	open/closed	MMC 15 mg/m^2^ CDDP 75 mg/m^2^	5 L saline; 60 min	41–42 °C	OS (2.5 years)	1. PFS; 2. M&M (30 d; 24 months) 3. MFS; 4. QoL (every 6 months)
NCT03348150	182	22 October	The Netherlands	CRS + HIPEC + sCTx	palliative sCTx	open	OX 460 mg/m^2^ DTX 50 mg/m^2^	ns; 30 + 90 min	41–42 °C + 37 °C	OS (5 years)	1. PFS 2. toxicity 3.cost and health benefits

MMC: mitomycin C; CDDP: cisplatin; MS: median survival; OS: overall survival; PFS: progression-free survival; QoL: quality of life; DTX: docetaxel; OX: oxaliplatin; sCTx: systemic chemotherapy; M&M: morbidity and mortality.

**Table 3:** Parameters of HIPEC in the included studies.

Reference	Before/After Anastomosis	Duration (min)	Open/Closed	Max. Heat (°C)	Drug First i.p. (mg/m^2^)	Drug Second i.p. (mg/m^2^)	Perfusate	Flow Rate	Number of Tubes	Bidirectional Drugs i.v.
Fujimoto et al. [29]	ns	120	ns	44.7–48.7	MMC 10 µg/mL; 30 mg td		3–5 L	ns	ns	
Fujimoto et al. [30]	ns	120	closed	43–45	MMC 10 µg/mL; 100 mg/L		3–4 L MWS	ns	2	
Chen et al. [31]	after	120	closed	40.5	MMC 30–40 m td		2–3 L RL	ns	2|2	
Sayag-Beaujard et al. [32]	ns	90	ns	46–49	MMC 10 mg/L		4–6 L	400–500 mL/min	2	
Loggie et al. [33]	ns	120	ns	40.5	MMC		ns	ns	ns	
Glehen et al. [34]	after	90	closed	46–48	MMC 10 mg/mL		4–6 L	500 mL/min	2|1	
Yonemura et al. [35]	after	60	open	42–43	MMC 30 mg td	td: CDDP 300 mg, Etoposid 150 mg	8 L saline	10 L/min	ns	
Kusamura et al. [36]	after	60–90	closed	42–43	CDDP 25 mg/m^2^/L	MMC 3.3 mg/m^2^/L	ns	ns	4	
Roviello et al. [37]	before	60	closed	41–43	MMC 25	CDDP 100	ns	700–800	5	
Scaringi et al. [38]	since 1998 before	90–120	o/c	41–43	MMC 120	CDDP 200	12 L saline	ns	2	
Yang et al. [39]	after	60–90	open	43 ± 0.5	HCPT 20 mg td	MMC 30 mg td	12 L saline	200 mL/min	1|1	
Piso et al. [40]	after	60	closed	42.5–43	CDDP 75	Doxorubicin 15	ns	ns	ns	
Yang et al. [41]	after	90–120	open	43 ± 0.5	td: HCPT 20 mg CDDP 120 mg	MMC 30 mg td	12 L saline	200 mL/min	1|1	
Li et al. [42]	after	60	closed	43 ± 1	CDDP 50 µg/mL	MMC 5 µg/mL	5–6 L	ns	2|1	
Glehen et al. [43]	ns	(1) 60–120 (2) 30 mean: 80.1	o/c	40–43; mean: 42.6	(1) MMC 30–50 (2) OX 360–460	(1) ±CDDP 100–200 (2) ±IRI 50–100	ns	ns	ns	5-FU + FA
Yang et al. [44]	after	60–90	open	43 ± 0.5	CDDP 120 mg td	MMC 30 mg td	6 L saline	500 mL/min	1|1	ns
Cotte et al. [45]	after	90	closed	44–46	MMC 0.7 mg/kg	IRI 100	3–4 L GLC	500 mL/min	2|1	
Mizumoto et al. [46]	after	60	ns	41–42	MMC 20 mg td	CDDP 100 mg td	saline	ns	2|1	
Wu et al. [47]	ns	60	ns	43 ±0.5	OX 460		3–4 L GLC	500–800 mL/min	3	
Yarema et al. [48]	after	90	open	43 ± 1.3	MMC 12.5	CDDP 75	ns	ns	ns	5-FU
Tabrizian et al. [49]	ns	60 + 30	closed	41–43	MMC †		ns	ns	ns	
Rudloff et al. [50]	before	30	closed	41	OX 460		3–4 L GLC	2 l/min	ns	
Magge et al. [51]	before	100	closed	42	MMC 30–40 mg td		3 L saline	800 mL/min	2|1	
Levine et al. [52]	after	60 + 60	closed	43	MMC †		3 L RL	1 L /min	2|2	
Kim et al. [53]	ns	60 + 30	ns	41	MMC †		ns	ns	ns	
Königsrainer I. et al. [54]	after	90	open	42	CDDP 50		ns	ns	ns	
Graziosi et al. [55]	after	60	closed	42	CDDP 25 mg/L/m^2^	MMC 3.3 mg/L/m^2^	ns	ns	ns	
Polanco et al. [56]	ns	ns	closed	42	MMC 40 CDDP 50		ns	ns	ns	
Desantis, M. [57]	before	60	open	43	CDDP 50		ns	800 mL/min	5	
Wu, H. T. [58]	before	60	open	43 ± 0.5	Lobaplatin 50	DTX 60	6 L saline	400 mL/min	ns	
Kopanakis [59]	ns	90	ns	ns	CDDP 50	Doxorubicin 50	ns	ns	ns	
Rihuete Caro [60]	after	90	open	42–43	CDDP 100	Doxorubicin 15	ns	ns	ns	
Montori et al. [61]	before	90	open	42–43	CDDP 100	Paclitaxel 175	ns	ns	1|4	
Yarema, R. [62]	ns	30–90	closed	42.7 ± 0.78	(1) MMC 10–15 (2) OX 460 (3) CDDP 75	(1) CDDP 75 (2) Doxorubicin 15	ns	ns	ns	5-FU
Solomon, D. [63]	before	90	closed	41–43	MMC 40 mg td		ns	ns	ns	
Rau [4]	after	60	o/c	41	MMC 15	CDDP 75	ns	ns	ns	
Manzanedo [64]	ns	ns	open		CDDP (50%)	Doxorubicin (50%)				
Kimbrough, C. W. [65]	ns	ns	o/c	ns	MMC		ns	ns	ns	
Hotopp [66]	ns	ns	open		OX 200	DTX 80	ns	1500 mL/min	ns	
Bonnot, P. E. [17]	ns	30–120	o/c	41–43	(1) MMC 30–50 (2) CDDP: 50–100 (3) OX: 300–460	(1) or (3) ±IRI 200 (1) +CDDP 100 (2) ±doxorubicin 15	ns	500 mL/min	ns	5-FU + FA
Braeuer, F. [67]	ns	45–60	closed	42	OX 400		ns	ns	ns	
Koemans, Willem J. [68]	before	30 + 90	open	41–42 (OX), 37 (DTX)	OX 460	DTX 0; 50, 75		ns	ns	

HCPT: hydroxycampthothecin; MMC: mitomycin C; CDDP: cisplatin; OX: oxaliplatin; IRI: irinotecan; DTX: docetaxel; ns: not stated; 5-FU: 5-fluorouracil; FA: folinic acid/leucovorin; td: total dose; MWS: Maxwell solutions-1S; RL: Ringer’s lactate; GLC: glucose 5%; o/c: open/closed; † 30 mg for the first, 10 mg for the latter perfusion duration; n1|n2 n1 inflow tubes|n2 outflow tubes.

**Table 4:** Overview of the extracted outcome parameters.

Reference	Year	Number of Patients with pmGC and CRS + HIPEC	Median PCI	Median OS	Clavien–Dindo ≥III°	Anastomotic Leakage Rate
Fujimoto et al. [29]	1988	9	ns	ns	ns	ns
Fujimoto et al. [30]	1997	30	ns	ns	ns	ns
Chen et al. [31]	1997	6	ns	ns	ns	ns
Sayag-Beaujard et al. [32]	1999	18	ns	ns	ns	ns
Loggie et al. [33]	2000	22	ns	ns	ns	ns
Glehen et al. [34]	2004	21	ns	10.3 months 1-year: 48.1% 2-year: 19.0% 5-year: 16.0%	13 (27%)	0/49 (0%)
Yonemura et al. [35]	2005	42	ns	11.5 months 5-year: 6.7%	ns	7/107 (6.5%)
Kusamura et al. [36]	2006	12	ns	ns	ns	ns
Roviello et al. [37]	2006	6	ns	ns	ns	ns
Scaringi et al. [38]	2008	26	ns	15 months	ns	ns
Yang et al. [39]	2009	12	ns	ns	ns	ns
Piso et al. [40]	2009	11	ns	ns	ns	0/15 (0%)
Yang et al. [41]	2010	28	12	1-year: 50.0% 2-year: 42.8%	ns	ns
Li et al. [42]	2010	10	ns	11.8 months 1-year: 52.5% 3-year: 13.2% 5-year: 5.5%	ns	0/10 (0%)
Glehen et al. [43]	2010	147	9.4 (±7.7)	9.2 months 1-year: 43% 3-year: 18% 5-year: 13%	34.30%	ns
Yang et al. [44]	2011	34	15	11 months 1-year: 41.2% 2-year: 14.7% 3-year: 5.9%	ns	0/35 (0%)
Cotte et al. [45]	2011	12	ns	ns	ns	0/12 (0%)
Mizumoto et al. [46]	2012	16	10 (±10) *	ns	38%	ns
Wu et al. [47]	2013	32	ns	15.5 months	ns	ns
Yarema et al. [48]	2014	20	3.40	12 ± 1.6 months	ns	1 (2%)
Tabrizian et al. [49]	2014	12	ns	3-year: 16.6%	ns	ns
Rudloff et al. [50]	2014	9	ns	11.3 months	8 (89%)	2 (22%)
Magge et al. [51]	2014	23	10.5	9.5 months 1-year: 49.6% 3-year: 17.9%	52%	3 (13%)
Levine et al. [52]	2014	46	ns	6.1 months	see Ref [53]	ns
Kim et al. [53]	2014	9	ns	16 months	ns	ns
Königsrainer et al. [54]	2014	13	ns	8.9 months	11 (Grade 1–5)	0 (0%)
Graziosi et al. [55]	2014	15	ns	ns	4 (11.1%)	0 (0%)
Polanco et al. [56]	2015	24	13	ns	see Ref [57]	ns
Desantis [57]	2015	14	ns	ns	ns	ns
Wu [58]	2016	50	15	24.8 months	12 (23.1%)	1 (1%)
Kopanakis [59]	2017	14	15	ns	ns	ns
Rihuete Caro [60]	2018	32	ns	ns	38%	ns
Montori et al. [61]	2018	26	8	16 months 1-year: 70.8% 3-year: 21.3%	9 (25.7%)	ns
Yarema [62]	2019	70	ns	PCI 0–6: 15 months PCI > 6: 8.2 months	ns	7 (6.5%)
Solomon [63]	2019	18	ns	12 months	see Ref [63]	ns
Rau [4]	2019	58	8.3 (±5.7) *	9.8 months	14 (62%)	ns
Manzanedo [64]	2019	84	6	21.2 months 1-year: 79.9% 3-year: 30.9%	30 (34.4%)	ns
Kimbrough [65]	2019	28	12	10 months	5 (18%)	2 (7%)
Hotopp [66]	2019	26	10	17 months	ns	2 (7.7%)
Bonnot [17]	2019	180	6	18.6 months	53.70%	ns
Braeuer [67]	2020	37	3.75 ±1.9 *	33.8 months	3 (37.5%)	ns
Koemans [68]	2021	23	2	15 months	ns	ns

* Mean (± standard deviation); OS: overall survival; ns: not stated.

## Data Availability

Data supporting reported results can be found in the cited literature, we did not include archived datasets.

## Supplementary Materials

The following supporting information can be downloaded at: https://www.mdpi.com/article/10.3390/jcm11051456/s1, Table S1: Reasons for exclusions.
