# Supplementary material for: HIPEC in Peritoneal Metastasis of Gastric Origin: A Systematic Review of Regimens and Techniques
Source: J Clin Med. 2022 Mar 7;11(5):1456. doi: 10.3390/jcm11051456 (PMC8911234; doi:10.3390/jcm11051456)
Supplement: Supplementary file 1 [file jcm-11-01456-s001.zip › jcm-1609095-SI.pdf]

Table S1: Detailed Reasons for exclusions **a:** Reasons for exclusion during abstract-screening; **b:** Reasons for exclusion during full-text-screening

a

Excluded during abstract-screening:
Conference abstract: 56
Review: 44
No pmGC patients: 20
Study Protocol: 19
Small cohort <6 pmGC patients: 15
Multiple injections of e.g. i.p. Paclitaxel: 13
Abstract not available: 11
No CRS: 11
Experimental study: 6
Neo-adjuvant: 6
EPIC: 5
Port: 5
Case Report: 3
Single injection: 2
Other: 12

b

Excluded during full-text-screening:
Full-text not available: 18
Insufficient info on HIPEC regimen: 13
Data included in other publication: 8
EPIC: 6
Small cohort <6 pmGC patients: 4
No CRS: 3
No pmGC patients: 1
Multiple injections of e.g. i.p. Paclitaxel: 1
Neo-adjuvant HIPEC: 1
